# The Imbalance in Medico-Legal Cover Awareness and Uptake Between Overseas Junior Doctors and Local Graduates in the NHS

**DOI:** 10.7759/cureus.13336

**Published:** 2021-02-14

**Authors:** Mustafa Jalal, Schaida Schirwani, Karna Dev Bardhan

**Affiliations:** 1 Department of Infection, Immunology and Cardiovascular Diseases, University of Sheffield, Sheffield, GBR; 2 Academic Department of Gastroenterology, Sheffield Teaching Hospitals, Sheffield, GBR; 3 Yorkshire Regional Genetics Service, Chapel Allerton Hospital, Leeds Teaching Hospitals NHS Trust, Leeds, GBR; 4 Gastroenterology, Rotherham Hospital, Rotherham, GBR

**Keywords:** overseas doctors, img, medico-legal insurance, medical defence organisation

## Abstract

Background

Recent reports showed that overseas doctors were more likely than UK graduates to be referred by their employers to the General Medical Council (GMC)*.* We investigated the trend of medico-legal insurance awareness and uptake of medical defence organisations (MDOs) by junior doctors and to examine if there is a difference between overseas and UK graduates.

Methods

Online questionnaire survey sent to junior doctors within the Yorkshire and Humber Deanery. Data regarding year of graduation, country of origin of primary medical qualification, year of starting work in the National Health Service (NHS) and date of joining an MDO were collected. Participant-identifiable information was not collected.

Results

A total of 202 junior doctors completed the survey: 153 (76%) UK graduates and 49 (24%) overseas. Overseas doctors were less likely to know about MDO compared to UK graduates prior to working in the NHS (13 [26.5%] vs. 146 [95.4%]; p < 0.0001). At the time of starting practice, MDO uptake was still significantly lower amongst overseas graduates (4 [8.2%] vs. 144 [94.1%]; p < 0.0001). Uptake by overseas doctors increased after starting work to 33 (67.3%). However, despite improvement in MDO uptake, a significant number of overseas doctors still did not have independent cover compared with UK graduates (16 [32.7%] vs. 3 [2%]; p < 0.0001).

Conclusions

Overseas graduates joining the NHS are still less likely to be aware of the requirement of adequate medico-legal cover and are less likely to join an MDO compared with UK graduates. Healthcare providers and regulators should work to decrease the existing gap and increase awareness amongst newly arrived overseas doctors.

## Introduction

The National Health Service (NHS) continues to be an attractive health system for doctors worldwide. Around 80,000 doctors qualified from 200 countries are licensed to work in the UK [1]. Overseas doctors, including international medical graduates (IMGs) and graduates from European Economic Area (EEA), represent one-third of the UK doctors’ workforce. Despite frequent changes to visa entry requirements and work permits, the NHS was able to retain a large proportion of overseas doctors; therefore, it is likely the demand on recruiting graduates from outside the UK will continue [2].

One of the challenges overseas doctors face is the lack of information on the NHS structure and culture [3]. The differences between the health system and the medical law between the country of origin and that of the UK may create a number of challenges during the transition into the UK [4]. Although there has been increasing effort to support overseas doctors in the NHS, the need for adequate medico-legal cover for them seems to be overlooked [5].

Registration with the General Medical Council (GMC) is mandatory for doctors working in the NHS and requires a standard of factual knowledge commensurate with experience. Fitness to practice requires doctors to have the appropriate skills, knowledge, health and character to perform their job effectively and safely. Also, it is expected that practicing doctors have, in addition to NHS Trusts’ indemnity scheme, adequate medico-legal insurance via independent medical defence organisations (MDO) to cover the scope of their practice [6]. Failure to arrange indemnity or failure to comply may result in withdrawal of licence and can be treated as misconduct in fitness to practice proceedings [7]. The GMC is responsible to assess doctor’s fitness to practice when a doctor is referred because of performance concern. Recent reports showed that overseas doctors were twice more likely to be referred by their employer and more likely than UK graduates to receive a warning or sanction [1,8].

Recently, there has been a call for introducing new and emergency laws to protect NHS doctors during the coronavirus disease 2019 (COVID-19) pandemic. Many doctors have to make difficult decision during this unprecedented time, including withholding or withdrawing treatment, which make them vulnerable to legal and regulatory investigation more than any other time [9].

There is lack of information about the adequacy of medico-legal cover for overseas doctors, and given the significant finding of the high proportion of overseas doctors referred to the GMC with potential harsh sanctions, we aimed to investigate the trend of MDO awareness and uptake by overseas junior doctors and to examine if there is a difference in awareness and uptake between overseas and UK graduates.

## Materials and methods

Design of online questionnaire and population

This is an online cross-sectional questionnaire survey designed using a dedicated website platform. The sample included junior doctors in training from foundation year 1 (FY1; first year of training) to final year specialist training; the duration of training can vary between specialities (Table 1).

**Table 1:** The questionnaire

Questions in the questionnaire
1. What is your grade?
2. Month and year of qualification?
3. Country of origin of primary medical qualification?
4. What is the date of start working in the NHS? Please specify month and year
5. Did you know about medical defence organisations before working in the NHS? Yes/No
6. Are you a member of a medico-legal organisation? Yes/No
7. If yes, date of joining? Please specify month and year
8. If you are a member, how did you hear about Medical Defence Organisations? (a) University; (b) Hospital induction; (c) Colleagues; (d) Friends; (e) Family; (f) others, please specify
9. Do you have any further comments?

The survey included junior doctors in training and non-training posts, whom we contacted by medical education and medical staffing departments. The person in these departments then contacted their junior doctors on our behalf. We approached junior doctors in 21 acute hospitals, across 15 NHS trusts, in the Yorkshire and Humber deanery. Each NHS Trust received a questionnaire, but we do not know how many were finally circulated. All responses were from junior doctors except for one from a consultant, which was excluded from analysis. A link to the survey alongside a cover letter was sent via email. A follow-up email was sent at four weeks with a final reminder. The survey platform prevented multiple entries from the same participant. Participation was voluntary, no incentive was offered and no participant-identifiable information was collected.

Statistical analysis

Data are presented as mean and standard deviation for continuous variables. Categorical data were analysed using Fisher’s exact test. A p-value of <0.05 was considered statistically significant. For ease of reading, percentages in the Results section are shown as whole numbers. Statistical analyses were performed using SPSS (IBM SPSS Statistics Version 25, IBM Corp., Armonk, NY, USA).

## Results

Cohort demographics

A total of 202 doctors completed the survey between February and July 2019: 153 (76%) UK graduates, 39 (19%) IMGs, 10 (5%) EEA. IMGs and EEA graduates were analysed as one group (overseas doctors) (n = 49 [24%]) (Table 2). The average time for completing a survey was 1 minute 15 seconds. Doctors were grouped into foundation trainees (the initial two years after graduation), specialist trainees (ST1 to ST8) and ‘Trust Grade’, a non-training post with varying in experience from the former senior house officer to the Registrar.

**Table 2:** Responses by grade and country of origin of primary medical qualification

Grade	Cohort, n (%)	UK, n (%)	Overseas, n (%)	p-Value
Foundation trainee	40 (19.8)	38 (24.8)	2 (4.1)	0.0008
Specialist trainee	141 (69.8)	104 (68)	37 (75.5)	0.4
Trust grade	21 (10.4)	11 (7.2)	10 (20.4)	0.02
Total number	202	153	49	

Foundation trainees were over-represented in the UK graduates group compared to overseas doctors (25% vs. 4%). The proportion of trust grade doctors was higher in overseas doctors (20% vs. 7%).

Awareness of medical defence organisations 

Before working in the NHS, overseas doctors were less likely to know about MDO compared to UK graduates (27% vs. 95%). The main source of information for UK graduates was during their time in medical school, whereas their counterparts from overseas learned of its need from colleagues, family members, friends and through hospital inductions (Table 3).

**Table 3:** Awareness of medical defence organisation and source of information MDO, medical defence organisation

	UK, n (%)	Overseas, n (%)	p-Value
Awareness of MDO prior to working in the NHS	146 (95.4)	13 (26.5)	<0.0001
Source of information			
University/medical school	140 (91.5)	0	<0.0001
Hospital induction programmes	7 (4.6)	6 (12.4)	<0.03
Colleagues	2 (1.3)	19 (38.8)	<0.0001
Friends	2 (1.3)	8 (16.3)	<0.0001
Family	1 (0.7)	2 (4.1)	0.09
Others	0	1 (2)	0.2
No response	1 (0.7)	13 (26.5)	

Medical defence organisation uptake 

MDO uptake at time of working in the NHS was significantly lower amongst overseas graduates compared to UK graduates (8% vs. 95%). After working in the NHS, the majority (59%) of overseas doctors joined an MDO compared to UK graduates (4%). At the time of the survey, 2% UK graduates did not have MDO cover compared to 33% overseas doctors (Table 4).

**Table 4:** Medical defence organisation cover uptake based on primary medical qualification MDO, medical defence organisation

	UK, n (%)	Overseas, n (%)	p-Value
MDO cover at time of working	144 (94.1)	4 (8.2)	<0.0001
MDO cover after joining the NHS	6 (3.9)	29 (59.1)	<0.0001
No MDO at time of survey	3 (2)	16 (32.7)	<0.0001
Time between working in the NHS and joining MDO (mean ± SD) months	-13.5 (30.4)	12.9 (16.7)	<0.0001

We did not collect information on gender from responses to examine a trend; however, we found a significantly lower uptake of an MDO in non-training post (Trust grade) compared to training posts (2/10 [20%] vs. 31/39 [79.5%]); p = 0.0009) amongst overseas doctors.

The mean time of uptake of MDO cover after working in the NHS was significantly different between UK graduates and overseas doctors (-13.5 vs. 12.9 months; p < 0.0001). The mean time for UK graduates is prefixed with a minus sign, indicating they joined MDO before working in the NHS.

## Discussion

To the best of our knowledge, this is the first study investigating awareness and uptake of MDO by junior doctors practising in the UK. Our study suggests that overseas doctors are less likely to be aware of the requirement of obtaining adequate indemnity. We also identified that overseas doctors were less likely to obtain MDO at the start or during their practice in the NHS compared to local graduates.

The Medical Act 1983 state that doctor who hold a licence to practice is responsible for making arrangement for adequate medico-legal insurance cover [7]. Failure to comply may result in disciplinary actions or misconduct during fitness to practice proceedings. Three out of four overseas doctors were unaware of MDO prior to working in the NHS, and a significantly lower proportion of overseas doctors joined MDO at the start their practice. Although the uptake increased during their work in the NHS, there was still a third of overseas doctors did not join an MDO at the time of survey. There is a lack of similar studies to compare with our results. A report commissioned by the GMC, to examine and to understand the regulatory health system of the top 10 countries overseas doctors have graduated from, found differences in health regulatory system of the examined countries and that of the UK [4]. We assume that the practice of medical indemnity cover may differ between country of origin and that of the UK, which make it challenging to compare.

Despite the steady rise in the number of medico-legal claims, the standard of healthcare in the UK has remained one of the best globally [10]. It is argued that a well-structured medico-legal system can improve standards by encouraging learning from claim cases [10]. Indemnity cover can also offer protection to practising doctors and their patients. However, the consequences can be significant not only to the organisation but also to the doctors referred. The impact on the doctor can result in serious health and financial consequences [11,12].

Lack of information and guidance for newly appointed overseas doctors on medico-legal cover requirements may explain the lower rate of MDO uptake by overseas doctors (8%) despite that a higher proportion (27%) were aware about MDO before working in the NHS. The source of information for local graduates was mainly during medical school, and they joined on average a year before working in the NHS in contrast to overseas doctors who have known about it from sources other than medical school. Another explanation for lower MDO uptake by overseas doctors is the significant variation in the regulatory framework in the individual doctor’s country of origin and that which applied in the UK; therefore, the way regulations are implemented may vary between countries [4].

The current COVID-19 pandemic is posing unprecedented challenges for the NHS. Doctors may have to make very difficult decisions while working outside usual field of practice. Lack of adequate medico-legal cover render overseas doctors to be vulnerable to medico-legal implications. The healthcare service providers have the duty to ensure that their doctors are appropriately indemnified; therefore, the message needs to be reinforced so that overseas doctors are well informed about the medico-legal requirement and the implications of having inadequate cover.

The study has a number of limitations. One of the limitations is the lack of information from consultants. We deliberately did not approach them as those who have been practicing for a long time may not accurately recall past events. We also did not approach doctors from the private sector, but we expect their number to be small; we also acknowledge that we covered only doctors in secondary care. We want to emphasise that it is not within the scope of this paper to examine the reasons why doctors join an MDO or otherwise but merely to examine the trend of differences between UK and overseas doctors. The proportion of UK and overseas graduates in our study was close to the registry data held by the GMC: 76% UK graduates in our cohort compared with 66% in GMC, 19% and 25% in IMG, respectively, and 5% and 9%, in EEA, respectively [1]; therefore, the authors consider that their study might reflect what is happening with other overseas doctors in the rest of the UK. Future research is needed to explore the reasons for overseas doctors not joining an MDO.

## Conclusions

Our study showed that there is an imbalance in medico-legal cover between overseas doctors and local graduates, as three-quarters of surveyed junior overseas doctors were unaware of the requirement of adequate medico-legal cover and a third had not joined an MDO at the time of survey.

The current status shows that overseas doctors are more likely to be referred to the GMC compared to their local counterparts; therefore, healthcare regulators should address this gap by increasing awareness and ensure that a newly appointed overseas doctor has adequate medico-legal insurance.
